# The NLR-related protein NWD1 is associated with prostate cancer and modulates androgen receptor signaling

**DOI:** 10.18632/oncotarget.1850

**Published:** 2014-03-26

**Authors:** Ricardo G. Correa, Maryla Krajewska, Carl F. Ware, Motti Gerlic, John C. Reed

**Affiliations:** ^1^ Sanford-Burnham Medical Research Institute, La Jolla, CA; ^2^ Current address: Dept. of Clinical Microbiology and Immunology, Sackler Faculty of Medicine, Tel Aviv University, Tel Aviv, Israel

**Keywords:** NLR, PDEF, AR, SRY, prostate

## Abstract

Prostate cancer (PCa) is among the leading causes of cancer-related death in men. Androgen receptor (AR) signaling plays a seminal role in prostate development and homeostasis, and dysregulation of this pathway is intimately linked to prostate cancer pathogenesis and progression. Here, we identify the cytosolic NLR-related protein NWD1 as a novel modulator of AR signaling. We determined that expression of NWD1 becomes elevated during prostate cancer progression, based on analysis of primary tumor specimens. Experiments with cultured cells showed that *NWD1* expression is up-regulated by the sex-determining region Y (SRY) family proteins. Gene silencing procedures, in conjunction with transcriptional profiling, showed that NWD1 is required for expression of PDEF (prostate-derived Ets factor), which is known to bind and co-regulate AR. Of note, NWD1 modulates AR protein levels. Depleting NWD1 in PCa cell lines reduces AR levels and suppresses activity of androgen-driven reporter genes. *NWD1* knockdown potently suppressed growth of androgen-dependent LNCaP prostate cancer cells, thus showing its functional importance in an AR-dependent tumor cell model. Proteomic analysis suggested that NWD1 associates with various molecular chaperones commonly related to AR complexes. Altogether, these data suggest a role for tumor-associated over-expression of NWD1 in dysregulation of AR signaling in PCa.

## INTRODUCTION

Prostate cancer (PCa) is the most common noncutaneous cancer affecting men worldwide. According to the United States Cancer Statistics (USCS), PCa had the highest incidence rate in U.S. men from 2003 to 2007 among lethal forms of cancer, and ranked second as a cause of cancer-related death in the same period. Remarkably, about 2.8 million men in USA are potentially living with this condition (more than 2 percent of the current male population over 15 years old), where approximately 240,000 new cases were diagnosed in 2012 [1].

PCa progression involves many alterations in prostate epithelial cells that lead, in some cases, to an androgen deprivation-resistant phenotype (castration-resistant PCa, CRPC), which relates to a poor prognosis for patients. Amplification of the gene encoding the androgen receptor (AR), which typically acts as a transcription factor upon binding to androgenic hormones, occurs in approximately 80% of the CRPCa cases, representing the most common genetic alteration in this type of cancer [1]. In fact, the majority of PCa specimens in the setting of relapse from hormonal therapy expresses AR and these cancers remain dependent on AR signaling for proliferation and survival in the presence of castrate levels of androgens [2, 3]. In this context, targeting AR signaling is still considered a critical component of the approach to therapy of metastatic PCa.

NLRs (NACHT and Leucine Rich Repeat domain containing proteins) constitute a major subfamily of innate immunity proteins [4, 5], mostly acting as cytosolic pattern recognition receptors (PRRs) involved in the detection of cytoplasmic pathogens (pathogen-associated molecular patterns, PAMPs) and endogenous cell injury signals (danger-associated molecular patterns, DAMPs) [6]. The recognition of activating ligands by NLRs initiates a variety of host defense pathways through the activation of NF-κB, stress kinases, interferon response factors (IRFs) and/or inflammatory caspases [7-10]. Dysregulation of NLR activities has been described in a variety of maladies, including chronic inflammatory diseases and cancer predisposition [11, 12]. For example, NOD1 (NLRC1) is among the NLR family members shown to possibly play a role in tumorigenesis. NOD1 stimulation induces apoptosis of MCF-7 breast carcinoma cells, and NOD1-deficient MCF-7 cells generate larger tumors when injected into immunocompromised mice [13]. Mice deficient in other NLR members, such as NLRP3 and NLRP12, have also been shown to be more susceptible to colitis-associated colon cancer [12, 14]. Polymorphisms of the NLR genes *NOD1 (NLRC1)* and *NOD2* (*NLRC2*) have been correlated with altered cancer risk. *NOD1* gene polymorphisms have been associated with a variety of cancer types, possibly due to the recognition by this innate immunity protein of ligands from *H. pylori* (etiologic agent in gastric cancer and MALT lymphoma), *C. trachomatis* (putative etiologic agent in ovarian cancer), *P. acnes* (possible causative agent in PCa) and *C. pneumonia* (plausible etiological agent in lung cancer) [15]. In addition, NOD1 and NOD2 have been shown to be fully operative in prostate epithelial cells and, in cooperation with TLRs, may elicit immune responses during PCa progression [16].

The NLR protein family is highly diverse across vertebrate species and invertebrate marine organisms, sharing structural similarity with plant disease-resistance (R) proteins involved in the hypersensitive response against plant pathogens [7, 17, 18]. The NLR protein structure is based on a C-terminal leucine-rich repeat (LRR) domain that is involved in recognition of conserved microbial patterns or other ligands; a centrally located nucleotide-binding NACHT domain that mediates self-oligomerization and is essential for NLR activation; and a N-terminal effector domain, which is responsible for the interaction with adaptor molecules that result in signal transduction [4, 7, 19, 20]. In humans, 22 NLRs have been described including 14 NLRPs with a PYRIN effector domain at the N-terminus, and 5 NLRCs whose effector domain is comprised of a caspase recruitment domain (CARD).

Based on genomic analysis of potential NLR family members found in other organisms, a newly conserved NACHT-domain protein with WD40 repeats has been identified in fish [21]. This protein, recently named as NWD1 (NACHT and WD repeat domain-containing protein 1), clustered with Apaf1 (Apoptotic peptidase activating factor 1), a cytoplasmic protein structurally similar to the NLRs that forms the apoptosome and mediates caspase-9 activation in the context of apoptosis induction [22]. The N-terminal region of NWD1 contains no motifs known from other proteins but, similar to Apaf1, it possesses WD40 repeats instead of LRRs at the C-terminus. Interestingly, this newly found fish protein has orthologs in both mice and humans [21], neither of which had been previously characterized. Therefore, here we investigate NWD1’s function and further correlate its expression as a potential PCa biomarker. Gene expression profiling and functional analysis suggest that NWD1 is involved in AR signaling and PCa progression, acting to stabilize steady-state AR protein levels in cells.

## RESULTS

### Expression profiling and protein characteristics of NWD1

**Figure 1:**
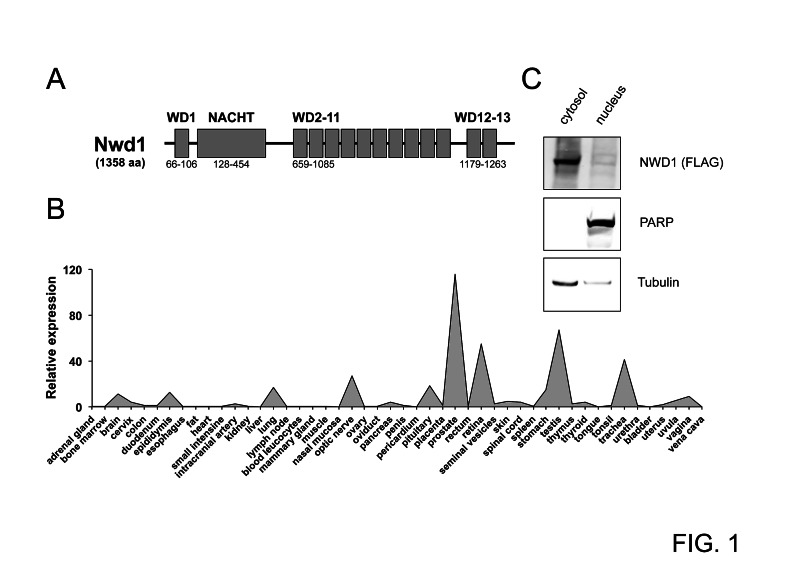
Characterization of NWD1 (A) Domain structure of NWD1. The protein contains a centrally located NACHT region and 13 WD40 repeats (WD1-13). Domain positions are indicated (bottom). (B) Tissue-specific expression of NWD1. A representative graph illustrating the relative *NWD1* expression in 48 independent human tissues is shown. First-strand cDNA derived from each tissue sample was normalized according to *GAPDH* expression (Human Major Tissue qPCR Array, HMRT102, Origene). (C) Subcellular localization of NWD1. HEK 293T cells cultured in a 10-centimeter plate to 90% cell confluency were transfected by calcium phosphate with 10 μg of FLAG-tagged NWD1 expression vector. After 48 hours, cells were fractionated. A total of 50 μg of respective nuclear and cytosolic extract fractions were resolved by SDS-PAGE, and further analyzed by immunoblotting using antibodies specific for α-tubulin (cytosolic marker), PARP (nuclear marker) and FLAG-epitope tag (for detection of ectopic NWD1).

The human *NWD1* gene spans about 98 kilobases (kb), and is located on chromosome 19p13.11 (NCBI Gene ID 284434). This gene is comprised by 19 exons (GenBank NM_001007525.3), and its transcript coding sequence is about 4 kb (position 689 to 4765 from GenBank BC117698.1). The *NWD1* gene is highly conserved among mammals, with over 70% nucleotide and amino-acid identities when comparing, for instance, human and murine orthologs. NWD1 was originally annotated as NACHT_P1, and listed as one NACHT domain-containing protein shared by both mammals and fish [21]. The NWD1 protein is phylogenetically clustered with Apaf1 (Apoptotic protease activating factor 1), due to the presence of WD40 repeats instead of LRRs typically found in NLR proteins. NWD1 consists of 1,358 amino acids (GenBank AAI17699.1) and has a calculated molecular mass of 150.84 kilodaltons (kDa). Its protein structure is composed of an N-terminal WD40 repeat, followed by a nucleotide-binding NACHT domain (amino acids 128-454) and then a WD40 repeat-containing region (12 repeats of about 40 amino acids each) at the C-terminus (Fig. 1A).

To access the tissue-specific gene expression profile for *NWD1*, quantitative PCR analysis was performed using a normalized cDNA panel derived from 48 human tissues (TissueScan Tissue qPCR Array, Origene). Two apparently independent expression patterns were detected, related to neurological organs (brain, pituitary and retina) and male reproductive system (prostate, epididymis and testis), where the highest mRNA levels were observed in prostate tissue (Fig. 1B). Results were confirmed using a second set of *NWD1*-specific qPCR primers (data not shown). Moreover, a similar expression pattern was virtually observed using the SAGE database from the Cancer Genome Anatomy Project (CGAP tag: AGTGACAGAG) of the National Cancer Institute (not shown).

The cellular localization of NWD1 protein was assessed by cell fractionation experiments, using human embryonic kidney (HEK) 293T cells transfected with a FLAG epitope-tagged NWD1 construct. As shown in Figure 1C, NWD1 was mainly detected in the cytosolic fraction, characterizing it as a potential cytosolic protein, similar to other NLR family proteins.

### SRY transcriptional factors are potential activators of *NWD1* expression

**Figure 2:**
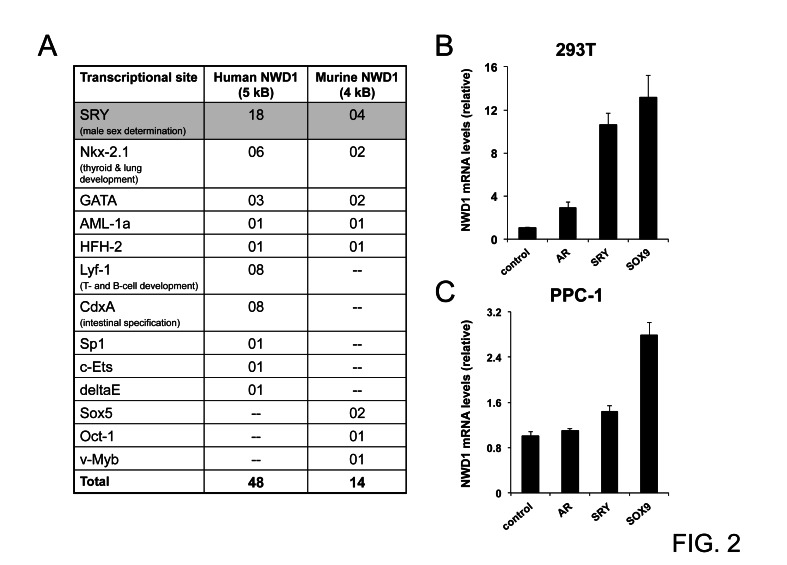
Upstream modulation of *NWD1* expression (A) Table listing predicted transcriptional binding sites (high scoring entries) conserved in murine and human *NWD1* promoter regions. Transcriptional sites were annotated using TRANSFAC™ matrix table with threshold of 95 (TFSEARCH™ version 1.3). The human and mouse promoter regions were estimated after BLAST analysis of respective genomic sequences (GenBank NC_000019.9, positions 16825787-16830786, and GenBank NC_000074.5, positions 75166647-75170613, respectively). The biological relevance of most representative sites is also described. (B) Real-Time PCR analysis of *NWD1* mRNA levels in transfected HEK 293T cells. Cells in 10-centimeter plates (90% cell confluency) were transfected by calcium phosphate with 10 μg of plasmid DNA encoding AR, SRY, SOX9 or EGFP (control). At 36 hours post-transfection, cells were processed for RNA purification and first-strand cDNA synthesis followed by PCR amplification. RNA levels were normalized by cyclophilin (CPH) expression. Relative values (y-axis) were obtained after comparison to expression in control cells (mean value = 1). Values presented are averages of at least three replicates (+ standard deviation). (C) Real-Time PCR analysis of *NWD1* mRNA levels in transfected PPC-1 cells. Cells in 10-centimeter plates (90% cell confluency) were transfected by Fugene HD™ (Roche) with 2 μg of respective DNA plasmids. At 48 hours after transfection, total RNA was isolated for first-strand cDNA synthesis and PCR amplification. RNA levels were normalized by 18S ribosomal RNA expression. Values were expressed as in (B).

To explore potential transcriptional modulators of *NWD1* expression, we virtually analyzed the content of high-scoring transcriptional sites located on the putative promoter regions of both human and mouse genes (5 kb and 4 kb of genomic 5’ regions, respectively), using TFSEARCH™ [23]. A few transcriptional sites were consistently enriched on both promoter regions (Fig. 2A), particularly SRY sites (sex-determining region Y). The SRY transcriptional sites are docking regions for members of the SOX (SRY-related HMG-Box) family of transcription factors, which are largely involved in male sex determination and neuronal development [24, 25]. This observation correlates with the determined pattern of *NWD1* gene expression in human tissues (Fig. 1B).

To validate the modulation of *NWD1* expression by SRY-related factors, HEK 293T and primary prostatic carcinoma (PPC-1) cell lines were transfected with constructs overexpressing the prototypical SRY (Sex-determining region Y protein) or the family counterpart SOX9 (SRY-Related HMG-Box, Gene 9) and then subjected to quantitative PCR analysis. *AR* over-expression was also analyzed since AR is known to interact with and to be modulated by SRY [26]. In 293T cells, both *SRY* and *SOX9* over-expression induced increases in NWD1 mRNA levels (10- and 13-fold increase, respectively) (Fig. 2B), while less striking increases were stimulated in PPC-1 cells (1.4- and 2.8-fold increase, respectively for SRY and SOX9) (Fig. 2C). The more limited induction of *NWD1* expression in PPC-1 cells was possibly due to the lower transfection efficacy when compared to HEK 293T cells. These results suggest that SRY transcription factors positively modulate *NWD1* expression. Further studies are required to determine whether SRY/SOX9 directly regulate *NWD1* expression under physiological conditions.

### NWD1 expression is increased in prostate tumorigenesis

**Figure 3:**
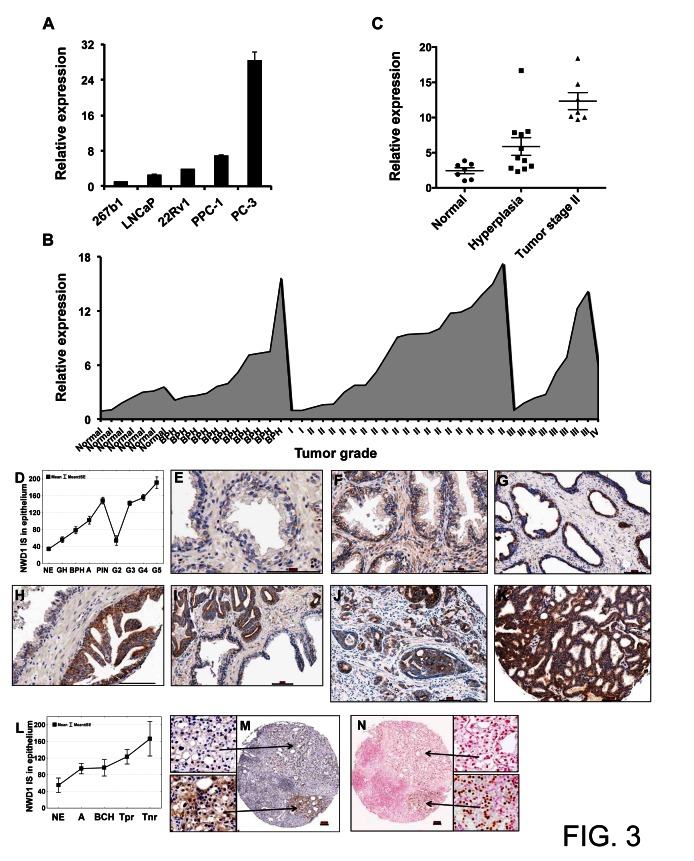
Expression patterns of NWD1 in prostate cancer (A) Real-Time PCR analysis of *NWD1* mRNA levels in a subset of prostate cell lines. RNA levels were normalized by cyclophilin (CPH) expression. Relative values (y-axis) were obtained after comparison to expression in normal prostate 267b1 cell line (mean value = 1). The values presented are averages of at least three replicates (+ standard deviation). (B) Stage-related expression of *NWD1* in prostate cancer. A limited sampling of four disease stages, benign prostate hyperplasia (BPH) and normal tissues was evaluated by Real-Time PCR analysis. A representative graph illustrating the relative *NWD1* expression using a pre-normalized cDNA panel, derived from respective tissue samples (n=48) at different tumor stages (TissueScan Prostate Cancer Tissue qPCR Panel I, HPRT101, Origene), is shown. (C) Comparative expression of *NWD1* in normal and hyperplasic (BPH) tissues and stage II tumors. Relative values derived from (B) were re-plotted for stage II tumor samples with assigned Gleason score and reported TNM (n=7). (D) NWD1 protein distribution in human prostate specimens. A box and whisker plot depicts NWD1 immunoscore data for normal prostatic epithelium (NE), glandular hyperplasia (GH), BPH, atrophy (A), prostatic intraepithelial neoplasia (PIN), and prostate adenocarcinoma Gleason grade 2-5 (G2–G5). (E-K) TMA sections containing prostate specimens from 109 prostate cancer patients were immunostained for NWD1 using polymer-based EnVision-HRP-enzyme conjugate and DAB chromogen (brown). Representative examples of NWD1 protein expression in the normal prostatic epithelium (E), BPH (F), atrophy (G), PIN (H), and progressing prostate adenocarcinoma (I-K) are provided. Specimens were counterstained with hematoxylin. Bar ~ 100 μm. (L) Immunohistochemical detection of NWD1 protein in castration-resistant prostate cancers. A box and whisker plot depicts NWD1 immunoscore data for normal prostatic epithelium (NE), atrophy (A), basal cell hyperplasia (BCH), and prostate cancer with partial (Tpr) or no response (Tnr) to androgen deprivation therapy. (M-N) Serial TMA sections containing 66 cores of prostate specimens from 18 prostate cancer patients with partial or no response to androgen deprivation therapy were immunostained for NWD1 (M) and AR (N) using polymer-based EnVision-HRP-enzyme conjugate and DAB chromogen (brown). Specimens were counterstained with hematoxylin (M) and nuclear fast red (N). Bar ~ 100 μm.

Due to the high *NWD1* expression detected in human prostate and its potential modulation by effectors involved in male sex differentiation and proliferation, we surveyed *NWD1* expression levels in a subset of prototypical PCa cell lines. The lowest expression levels were detected in the neonatal human prostate epithelial cell line 267b1, followed by a modest increase in the androgen-responsive and PSA (prostate-specific antigen)-expressing LNCaP and 22Rv1 cells (Fig. 3A). The highest levels of *NWD1* expression were observed in the androgen-independent and highly metastatic prostate cell lines PC-3 and PPC-1 (Fig. 3A).

Changes in the levels of *NWD1* expression, and its possible correlation with PCa progression, were further investigated by quantitative PCR analysis using a normalized cDNA panel derived from isolated prostate samples (n=48) at different tumor stages (TissueScan Prostate Cancer Tissue qPCR Panel I, Origene) (Fig. 3B). An increase in *NWD1* expression could be observed, particularly when comparing normal tissues (n=7) versus stage II tumor samples (n=19) (Fig. 3B). In fact, stage II tumor samples with assigned Gleason score and reported TNM (n=7) showed an increase of 5-fold (p<0.0001) in *NWD1* mRNA expression levels when compared to normal prostate tissues (Fig. 3C). An increase of 2.4-fold was also detected in hyperplasic tissues (BPH) (n=11) but failed to reach statistical significance when compared to normal samples (p=0.05) (Fig. 3C). A relative increase in NWD1 expression at the mRNA level could also be inferred for stage III prostate tumors but due to the limited number of samples tested (also for stages I and IV), no statistical difference could be assumed.

To verify the consistency of the RNA expression data with the respective protein yields, we generated polyclonal antibodies against NWD1 to further evaluate protein levels in prostatectomy samples containing tumor tissue representing different Gleason grades. The antibody specificity was validated by testing against other NLR family proteins (Supplementary Fig. 1). In prostate tissue specimens derived from 109 PCa stage T2 and T3 patients, immunohistochemical analysis revealed low expression of NWD1 in normal prostatic epithelium (n=39), which markedly increased in glandular hyperplasia (GH; n=28) (*p*=0.006), benign prostatic hyperplasia (BPH; n=40) (*p*<0.0001), atrophy (A; n=53) (*p*<0.0001), and prostatic intraepithelial neoplasia (PIN; n=48) (*p*<0.0001). Although barely expressed in Gleason grade G2 adenocarcinoma (n=11), NWD1 was significantly up-regulated in G3 (n=166) (*p*<0.0001), G4 (n=68) (*p*<0.0001), and G5 (n=8) (*p*<0.0001) PCa specimens compared to the normal prostate glands (Figs. 3 D-K). Increased levels of NWD1 immunostaining were found in samples of prostate tumors resistant to androgen deprivation therapy (n=20). Specimens with histologic evidence of partial (Tpr; n=14) or no response (Tnr; n=6) to pharmacological castration showed significantly elevated NWD1 protein levels compared to the normal prostatic epithelium (*p*=0.02) (Figs. 3 L-N). High expression of NWD1 (median cut-off) was associated with areas of AR positive immunostaining in 75% of hormone refractory tumor samples (15/20) (Figs. 3 M-N). Altogether, NWD1 can be considered as a potential PCa biomarker, since its increased expression at both RNA and protein levels is associated with malignant progression.

### NWD1 activity in androgen-independent prostate cancer cells

**Figure 4:**
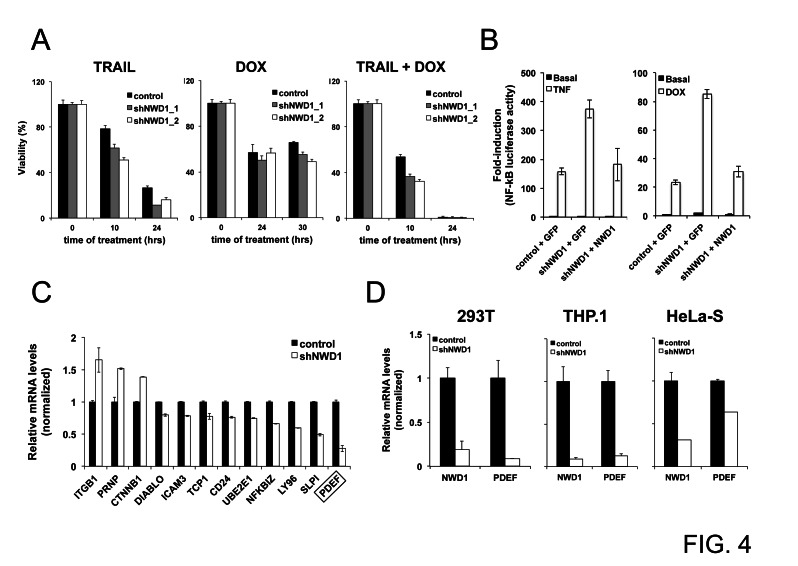
Effects of *NWD1* gene silencing *in vitro* (A) Cell viability analysis. PPC-1 cells (5x10^3^ cells/well in 96-well plate) constitutively expressing shRNAs against EGFP (control) or NWD1 (shNWD1_1 and shNWD1_2) were respectively treated with 100 ng/ml TRAIL, 1μM DOX or their combination for different times (10, 24 or 30 hours). Cell viability and growth was determined by performing WST-1 assay (Roche) reading absorbance at 450 nm (reference wavelength of 690 nm) according to the manufacturer’s instructions. Values were converted to percentage of viability according to the measurements obtained for non-treated cells (considered as 100% viable). The values presented are averages of four replicates (+ standard deviation). (B) Activity towards NF-κB signaling pathway. HEK 293T cells stably containing an NF-κB-mediated luciferase reporter gene (HEK 293T-κB) and constitutively expressing shRNA for GFP (control) or NWD1 (shNWD1) were plated in 10-cm dish and, at 90% confluency, were transfected by calcium phosphate with 20 μg of GFP or NWD1 expression vectors. At 24 hours post-transfection, cells were resuspended and loaded into 96-well plate at 5x10^4^ cells/well. Cells were further treated with 10 ng/ml TNF (left graph) or 0.4 μg/ml DOX (right graph) in serum-free medium for 18 hours. Luciferase activity was measured using Steady-Glo™ reagent (Promega). Values were converted to fold-induction in NF-κB activity according to the measurement obtained for non-treated (basal) control cells (mean value=1). Values represent averages of three replicates (+ standard deviation). (C) Real-Time PCR validation of putative NWD1 target genes identified by microarray analysis. PPC-1 cells constitutively expressing shGFP (control) or shNWD1 were processed for isolation of total RNA and first-strand cDNA synthesis. RNA levels were normalized by 18S ribosomal RNA expression. Relative values (y-axis) were obtained by comparison to the expression in control cells (mean value = 1). The values presented are averages of at least three replicates (+ standard deviation). The target gene with highest fold change on expression (PDEF) is indicated. (D) Real-Time PCR validation of PDEF as an NWD1 target gene in different cell lines. HEK 293T, THP.1 and HeLa-S cells constitutively expressing shGFP (control) or shNWD1 were respectively processed for isolation of total RNA and first-strand cDNA synthesis, followed by PCR amplification. RNA levels were normalized by cyclophilin (CPH) expression. Normalized levels of *NWD1* expression are also shown. Values were expressed as in (C)

To explore a functional role for NWD1 in PCa progression, we designed a lentiviral-based gene silencing approach to knockdown *NWD1* expression in PPC-1 cells (Supplementary Fig. 2), one of the PCa cell lines with high NWD1 mRNA levels (Fig. 3A). PPC-1 is a highly transfectable variant of PC-3 cells, originally described as a good model system for the study of PCa progression [27]. Under basal conditions, no major changes in cell proliferation or cell migration *in vitro* (wound healing assay) or tumor development in mouse xenografts (using 7 week-old female nude mice) were noticed when silencing *NWD1*, using PPC-1 as cellular model (data not shown). Also, PPC-1 cells treated with TRAIL (TNF-Related Apoptosis-Inducing Ligand), separately or in combination with the chemotherapy drug doxorubicin (DOX), were only slightly more sensitive when *NWD1* expression was silenced (Fig. 4A). No significant differences were noticed after treatment with the chemotherapy drug Taxol/Paclitaxel (data not shown).

With respect to affects of NWD1 gene silencing on cytokine signaling, we used a NF-κB dependent luciferase reporter as an assay platform, which is strongly activated by cytokines such as TNF-α and by the chemotherapeutic drug DOX. We observed that *NWD1*-depleted 293T cells produce higher NF-κB reporter gene activation after treatment with these inducers when compared to the control cells (Fig. 4B). Importantly, reconstituting *NWD1* expression in the shRNA-based depleted cells reduced NF-κB activation comparably to control levels (Fig. 4B). In contrast to NF-κB, experiments using an AP-1-driven luciferase reporter gene revealed no defect in AP-1 signaling following *NWD1* knockdown (Supplementary Fig. 3), thus demonstrating the specificity of our results. These observations indicate that *NWD1* expression can affect cell viability and NF-κB signaling, similar to many NLR family proteins [18, 28].

The participation of NWD1 in signaling transduction pathways was further examined by microarray analysis (Human WG-6v3 Expression BeadChip, Illumina), comparing the expression profile of PPC-1 cells lacking *NWD1* expression (~5% residual expression, Supplementary Fig. 2) versus control cells. According to the differential expression profile that was generated and analyzed by the Ingenuity Pathways™ software (IPA version 7.1, Ingenuity Systems), NWD1 is presumably associated with biological networks related to tissue morphology, organogenesis, cancer and neurological diseases (Supplementary Figs. 4-5). A list including most of the genes that are more dramatically up or down regulated after *NWD1* knockdown is shown (Supplementary Fig. 4). Still, a very limited number of genes was transcriptionally induced or repressed with a difference of > 2-fold (a total of 13 and 18 genes, respectively).

After validation of several promising hits by quantitative PCR analysis (Fig. 4C), we observed that *PDEF* was the most quantitatively down-modulated target gene, with approximately 4-fold reduction (~75% suppression). Interestingly, shRNA-mediated silencing of *NWD1* expression also reduced *PDEF* mRNA levels in other human cell lines, including 293T, THP.1 and HeLa-S cells (Fig. 4D), suggesting conservation of NWD1-dependence for *PDEF* expression across cell types.

PDEF is a prostate epithelial-specific Ets transcription factor, which is involved in PSA gene regulation and acts as a co-regulator of AR [29]. Remarkably, PDEF has been described as one of the few transcription factors with potential to have a significant impact on the management of PCa [30]. Since the expression of *PDEF* is typically up-regulated following activation of the AR signaling [31, 32], we considered that NWD1 might have a more intimate role on this signaling pathway and designed experiments to test this hypothesis.

### NWD1 activity in androgen-responsive prostate cancer cells

**Figure 5:**
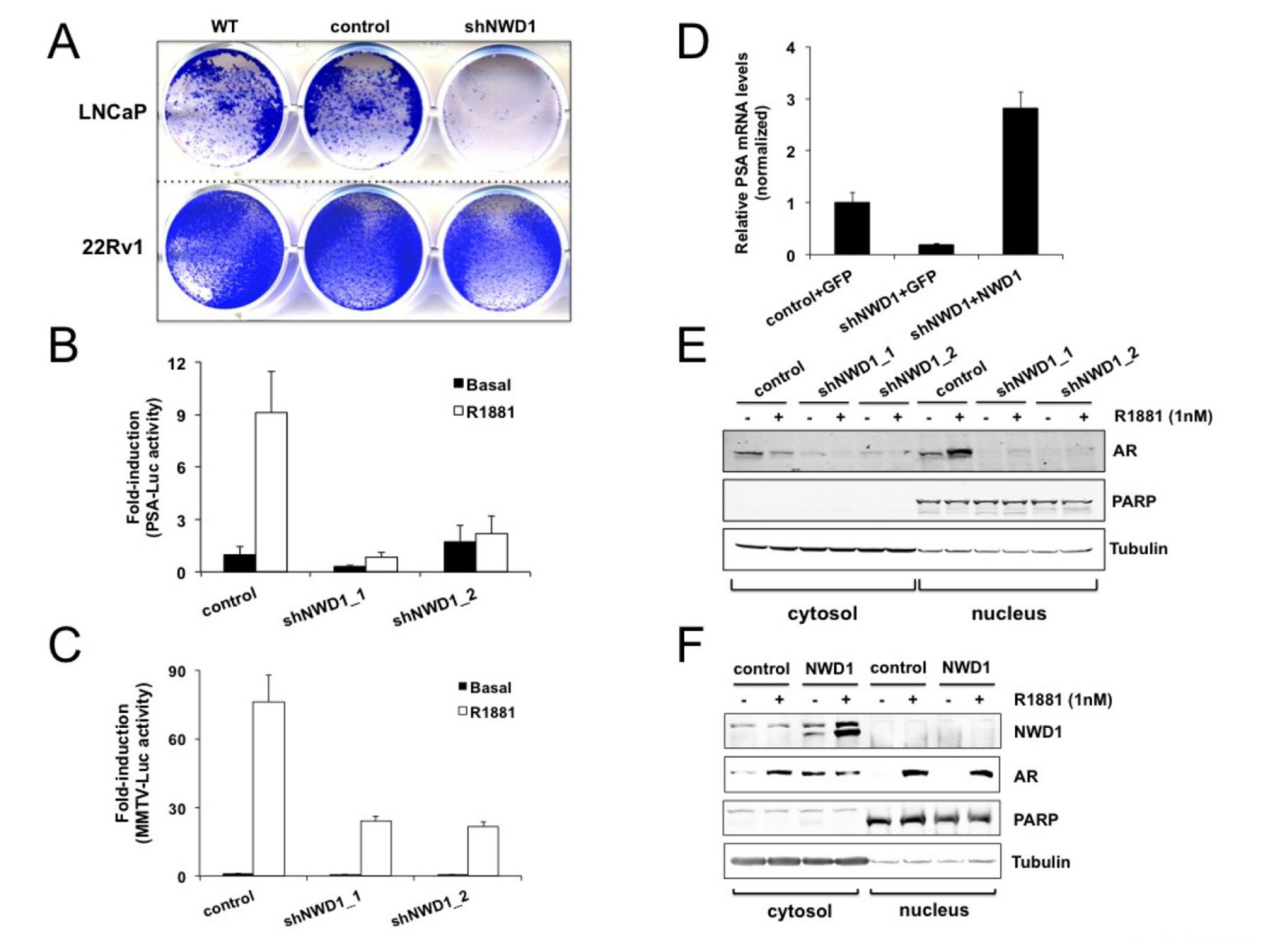
NWD1 participates in androgen receptor (AR) signaling (A) Viability analysis of androgen-responsive prostate cancer cells (LNCaP and 22Rv1). Cells plated in 6-well dishes at 90% confluency were infected with lentiviruses (MOI ≥ 100) to constitutively express shGFP (control) or shNWD1. At 48 hours post-infection, cells were transferred to 12-well dishes (10^5^ viable cells/well) and cultured for an addtional 3-4 days, before fixing cells and staining with crystal violet. Staining of non-infected wild-type (WT) cells was utilized as a standard control. (B) PSA-mediated luciferase reporter assay. LNCaP cells (10^6^ cells/well in 6-well dish) were transfected by GenJet reagent (SignaGen) with 1 μg of shGFP (control) or shNWD1 constructs plus 0.7 μg PSA-mediated firefly luciferase and 0.3 μg thymidine kinase (TK)-mediated Renilla reporter plasmids per well. After 24 hours of transfection, cells were transferred to 96-well dish (2×10^4^ viable cells/well in charcoal-stripped serum containing medium) and treated or not with 1 nM R1881 for 60 hours. Luciferase activity was measured using Dual-Luciferase™ Reporter Assay System (Promega). Values were normalized according to Renilla luciferase signals. Values were converted to fold-induction in PSA-mediated activity according to the measurement obtained for non-treated (basal) control cells (mean value=1). The values presented are averages of three replicates (+ standard deviation). (C) MMTV-mediated luciferase reporter assay. LNCaP cells were plated in 96-well dishes (3×10^4^ cells/well in charcoal-stripped serum containing medium) and transfected by JetPrime reagent (PolyPlus) using 0.05 μg of respective shRNA constructs, 0.02 μg of AR expression vector, 0.02 μg MMTV-mediated firefly luciferase and 0.01 μg thymidine kinase (TK)-mediated Renilla reporter plasmids per well. After 24 hours of transfection, cells were induced or not with 1 nM R1881 for further 24 hours. Luminescence values were measured and processed as in (B). (D) Real-Time PCR analysis of *PSA* expression levels. HEK 293T cells in 10-centimeter plates (90% cell confluency), constitutively expressing shGFP (control) or shNWD1, were transfected by calcium phosphate with 20 μg of DNA constructs to respectively overexpress GFP or NWD1. At 40 hours post-transfection, cells were processed for standard RNA purification and first-strand cDNA synthesis. RNA levels were normalized by cyclophilin (CPH) expression. Relative values (y-axis) were obtained by comparing to the *PSA* expression in GFP-transfected control cells (mean value = 1). The values presented are averages of at least three replicates (+ standard deviation). (E) Analysis of exogenous AR content and trafficking by subcellular fractionation. PPC-1 cells in 10-centimeter plates (90% confluency) were transfected by JetPrime reagent (PolyPlus) with 6 μg of shRNA constructs to knockdown EGFP (control) or NWD1 (shNWD1_1 and shNWD1_2) plus 3 μg of AR expression vector. Cells were split 24 hours after transfection and cultured in charcoal-stripped serum containing media for 48 hours. Cells were further treated with (+) or without (−) 1 nM R1881 for 28 hours. Cytosolic and nuclear subcellular fractions were isolated and analyzed (50 μg protein) by immunoblotting using antibodies specific for α-tubulin (cytosolic marker), PARP (nuclear marker) and AR. (F) Analysis of endogenous AR content and trafficking by subcellular fractionation. LNCaP cells in 10-centimeter plates (70% confluency in charcoal-stripped serum containing medium) were transfected by JetPrime reagent (PolyPlus) with 10 μg of GFP (control) or FLAG-NWD1 expression constructs. At 48 hours post-transfection, cells were treated with (+) or without (−) 1nM R1881 for 32 hours. Fractionation analysis followed as in (E), including anti-FLAG antibody for exogenous NWD1 detection.

To investigate a potential role for NWD1 in AR signaling in PCa, we stably knocked down expression of *NWD1* in androgen-responsive PCa cells LNCaP and 22Rv1. Strikingly, constitutive depletion of *NWD1* expression dramatically affected the viability of transduced LNCaP cells, whereas the same depletion marginally affected the proliferation of transduced 22Rv1 cells (Fig. 5A). A consistent decrease in cell viability was observed using two additional lentiviral-based shRNA constructs targeting *NWD1* (data not shown). Despite both cells lines being androgen-responsive, androgen-dependent cell proliferation is only observed for LNCaP cells. In fact, 22Rv1 cells express two separate AR protein species, where one of them is constitutively nuclear and binds DNA independent of androgens [33], accounting for why its proliferation is less affected by hormone ablation. Decreased cell proliferation and reduced cell viability were also observed in NWD1 shRNA-transduced LAPC-4 cells, another androgen-responsive cell model (data not shown). These observations suggest that NWD1 could be important for AR activity and/or downstream AR-mediated signaling events in androgen-dependent PCa cells.

To evaluate whether NWD1 depletion could modulate AR signaling in androgen-dependent prostate cells, we transiently knocked down *NWD1* expression in LNCaP cells and then performed reporter gene assays to monitor effects on either the PSA or MMTV promoters, both known to be activated by androgens (Figs. 5 B-C). Under basal conditions, no major differences were noticed regarding PSA- or MMTV-mediated luciferase reporter gene activity. In contrast, androgen-dependent luciferase activity induced by treatment with the synthetic androgen R1881 was consistently attenuated by shRNA-mediated silencing of *NWD1* expression. PSA-dependent luciferase activity in androgen-treated cells was reduced by 76 to 91% after *NWD1* silencing (using two independent shRNA constructs) (Fig. 5B). Similarly, MMTV-dependent luciferase activity in androgen-treated cells was diminished by approximately 70% as a result of *NWD1* gene silencing (Fig. 5C). In contrast to gene knockdown, *NWD1* over-expression did not lead to major changes in PSA- or MMTV-mediated luciferase activities (data not shown). Based on these results, we conclude that NWD1 potentially participates in AR signaling, since its depletion negatively affects androgen responsiveness towards the activation of AR-responsive reporter genes.

Next, we evaluated the impact of stable and constitutive *NWD1* gene silencing on expression of the endogenous *PSA* gene, a prototypical AR target gene [34]. Previously, it has been reported that HEK cells can recapitulate AR biochemical activity [35], thus providing a convenient context for undertaking expression studies. PSA mRNA levels were substantially decreased in NWD1-depleted HEK 293T cells, showing approximately 5-fold reduction in *PSA* expression when compared to control cells (Fig. 5D). Reconstitution of these deficient cells by *NWD1* over-expression restored *PSA* transcript levels that, in fact, resulted in an increase of approximately 3-fold over control cells (Fig. 5D). This result confirms NWD1’s role in modulating *PSA* expression, despite the fact that *AR* is rarely expressed in 293T cells.

The activity of AR is impacted by multiple mechanisms, including regulation of protein stability, cellular trafficking (cytosol-nuclear translocation), post-translational modifications, and interactions with co-activators, co-repressors, and various chromatin-modifying proteins [36, 37]. To preliminarily probe the mechanisms by which NWD1 modulates AR activity, we assessed the impact of *NWD1* knockdown on AR protein levels in PCa cells, obtaining evidence of a decrease in AR levels in NWD1-deficient cells. For example, in experiments using AR-overexpressing PPC-1 cells, *NWD1* knockdown was associated with reduced ectopic levels of both cytosolic and nuclear AR protein, both in the presence and absence of the AR agonist R1881 (Fig. 5E). In addition, endogenous AR levels were also decreased in 22Rv1 prostate cells stably expressing NWD1 shRNA (Supplementary Fig. 6A). This decrease in AR protein is not due to ubiquitination and proteasomal degradation, since treatment of cells with the proteasome inhibitor MG-132 does not restore AR protein levels (Supplementary Figs. 6A-B). In fact, in accordance with literature reports [37], proteasome inhibition was associated with reduced AR protein expression (e.g., a dramatic decrease of AR endogenous levels was observed in 22Rv1 cells while no major effect was observed on the ectopic levels of AR-overexpressing PPC-1 cells [Supplementary Fig. 6]).

While *NWD1* knockdown caused a reduction in AR protein levels, conversely, *NWD1* over-expression increased AR endogenous levels in PCa cells. In LNCaP cells, *NWD1* over-expression resulted in higher AR basal levels in the cytosol compared to control, when cells were cultured without androgens (Fig. 5F). No apparent changes in the levels of AR translocated to the nucleus were observed after *NWD1* over-expression (Fig. 5F) This direct correlation between NWD1 and AR protein levels is consistent with our observations in PCa tumor samples (Figs. 3 M-N). We conclude therefore that NWD1 controls steady-state levels of AR. We presume that this accounts at least in part for the ability of NWD1 to modulate AR bioactivity, but cannot exclude the possibility of additional causes such as an impact on AR subcellular trafficking, post-translational modifications, or association with chromatin-modifying proteins.

To gain additional insights into the mechanisms by which NWD1 may modulate AR activity, we sought to identify NWD1 interacting proteins. To this end, we performed LC-MS/MS proteomic analysis of FLAG-tagged NWD1 immunoprecipitates (IPs) isolated, at high stringency, from androgen-stimulated LNCaP cells. After subtractive analysis, using FLAG-tagged EGFP IPs as a control, a limited number of interacting proteins were identified, which included chaperone-related proteins such as Hsp90, Hsp40, Bag-2, TRAP1/Hsp75 and HSPA1B (Table 1). Some of these interacting molecules, including Hsp90 and Bag-2, were validated by independent immunoprecipitation assays (Supplementary Fig. 7). Since no evidence of direct binding between NWD1 and endogenous AR was obtained (data not shown), we hypothesize that NWD1 could affect AR activity through the interaction with chaperone complexes.

**Table 1:** NWD1 interacting proteins identified by proteomic analysis. LNCaP cells in T-75 flasks (70% confluency), cultured in standard RPMI 10% FBS supplemented with 0.5 nM R1881 (to stimulate cell growth), were transfected by TransIT-2020 reagent (Mirus) with 10 μg of FLAG-GFP (control) or FLAG-NWD1 expression constructs. At 96 hours post-transfection, cells were lysed for further immunoprecipitation (IP) procedures using anti-FLAG beads. Mass spectrometry (1D LC-MS/MS) was performed twice using respective IP eluates. Protein hits also present in the control sample were subtracted to point out specific NWD1-related hits. Each protein entry shows the respective IPI (International Protein Index) number and the total protein spectral counts. The respective protein functions are also listed.

IPI#	Gene Symbol	Protein Identified	Spectral Count	Function
IPI00744647	NWD1	NACHT and WD repeat domain-containing protein 1	401	
IPI00007752	TUBB2C	Tubulin beta-2C chain	99	microtubule structure
IPI00941328	HSPA1B	Heat shock 70kDa protein 1B	92	chaperone
IPI00382470	HSP90AA1	Heat shock protein HSP 90-alpha	54	chaperone
IPI00414676	HSP90AB1	Heat shock protein HSP 90-beta	50	chaperone
IPI00292496	TUBB8	Tubulin beta-8 chain	42	microtubule structure
IPI00015947	DNAJB1	DnaJ (Hsp40) homolog subfamily B member 1	22	co-chaperone
IPI00008868	MAP1B	Microtubule-associated protein 1B	21	microtubule assembly
IPI00000643	BAG2	BAG family molecular chaperone regulator 2	11	co-chaperone
IPI00480014	SSX11	Synovial sarcoma X (SSX) breakpoint protein 11	10	transcriptional modulation
IPI00030275	TRAP1	Heat shock protein 75 kDa, mitochondrial	8	chaperone
IPI00000690	AIFM1	Apoptosis-inducing factor 1, mitochondrial	7	regulation of apoptosis
IPI00020058	ATP7B	Copper-transporting ATPase 2	4	copper transport
IPI00554648	KRT8	Keratin, type II cytoskeletal 8	4	cytoskeleton structure
IPI00015842	RCN1	Reticulocalbin-1	3	calcium binding
IPI00021187	RUVBL1	RuvB-like 1	3	transcriptional modulation
IPI00029628	RCN2	Reticulocalbin-2	3	calcium binding
IPI00064328	PRMT5	Protein arginine N-methyltransferase 5	3	metabolic modulation
IPI00440493	ATP5A1	ATP synthase subunit alpha, mitochondrial	3	ATP synthesis
IPI00032406	DNAJA2	DnaJ (Hsp40) homolog subfamily A member 2	2	co-chaperone
IPI00909055	KLHDC3	Kelch domain-containing protein 3	2	meiotic recombination

**Figure 6:**
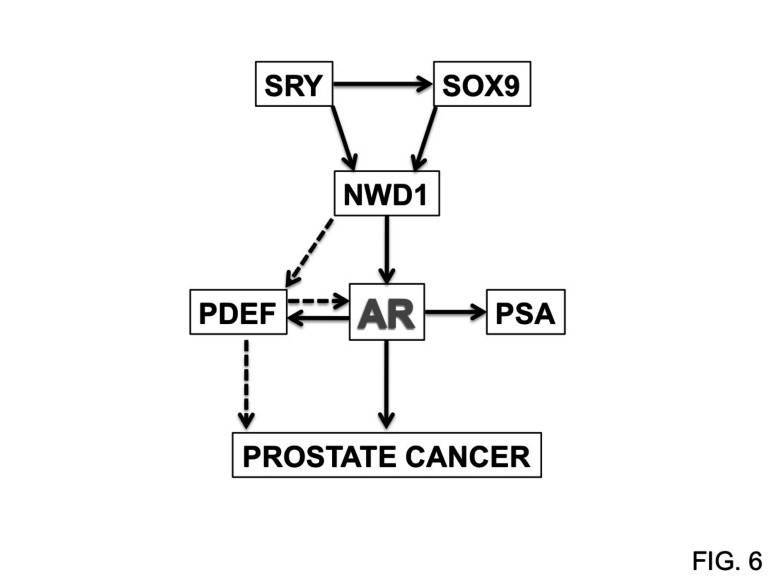
NWD1-dependent AR signaling Model depicting the participation of NWD1 in AR signaling and its putative correlation with prostate cancer progression. Dashed arrows indicate sub-pathways that require further investigation.

## DISCUSSION

Here we characterize NWD1 as a newfound NLR-related protein that contains a central NACHT domain akin to classical NLR family members but that lacks the LRRs required for formal membership in the NLR family. In contrast to mammals, a large number of “atypical” NLRs, containing altered domain organizations have been described in cnidarians [39] and fish [21, 38, 40]. To our knowledge, NWD1 is a unique variant among the NLR members found in humans, since it contains the NACHT domain in the absence of accompanying LRRs. NWD1 possesses WD40 repeats flanking the central NACHT domain, and thus it resembles to some extent the pro-apoptotic protein Apaf1. The LRR regions of NLRs have a seminal role in pathogen sensing, whereas the WD40 repeats are generally known to be involved in intracellular protein–protein interactions. For example, the WD40 repeat region of Apaf1 promotes binding to cytochrome c to trigger formation of the apoptosome and thus initiate caspase activation leading to apoptosis [41].

The human *NWD1* gene is located on chromosome 19p13.11, a genomic region that has shown susceptibility to genomic alterations in hormone-related cancers (ovarian, breast and prostate) [42-45]. In fact, among eighteen loci associated with more than one hormone-related cancer, this region was particularly identified as a breast and ovarian cancer susceptibility locus by the COGS (Collaborative Oncological Gene-environment Study) consortium [43-45]. Other pathological conditions, including CNS diseases such as ASD (autism spectrum disorder) [46] and pontocerebellar hypoplasias (PCHs) [47], have shown copy number variations and *de novo* deletions linked to the 19p13.11 locus, suggesting that this region might contain genes that could be important for CNS development. Interestingly, though not as prominent as male sex hormone-dependent tissues, we found that tissues such as brain, pituitary, optic nerve and retina were among those with higher *NWD1* expression.

Expression analysis along a variety of human tissues indicate that male reproductive organs including epididymis, testis and particularly prostate, represent the organs with the highest NWD1 mRNA levels. Interestingly, the expression of some NLR family members, including NLRP2, -3 and -10, was previously detected at high levels in reproductive organs [48]. Indeed, mutations in the NLR gene *NLRP7* has been described as a causative hallmark for recurrent hydatidiform moles (RHM) and reproductive loss [49].

A potential correlation of NWD1 with prostate development was also inferred based on prediction of tissue-specific transcriptional factors that potentially bind within the putative promoter region of the *NWD1* gene. In this regard, inspection of the suspected promoter regions of both human and mouse orthologous genes indicated that the most represented potential cis-regulatory sites were consensus DNA binding sites for SRY/SOX9. SRY is a testis-expressed protein that functions as a high mobility group (HMG) box transcription factor and is encoded by the sex-determining region on the Y chromosome that mediates male sex determination in mammals [50]. Translocations of *SRY* into the X chromosome (during paternal meiosis) cause XX individuals to be male, and loss of function mutations of *SRY* cause XY individuals to be female [24]. *SRY* expression has also been reported in prostatic tissues, and loss of *SRY* and other Y-chromosome-encoded genes is a frequent finding in prostate cancer [51-53]. The genes downstream of SRY involved in sex determination are encoded by autosomes, including the major target sex-determining region Y box 9 (*SOX9*). SRY binds to multiple gonad-specific enhancer elements of the *SOX9* promoter to up-regulate its expression [50]. High levels of SOX9 have been reported to modulate epithelial cell proliferation within the prostate gland and to cooperate with mutations such as *PTEN* loss to accelerate prostate neoplasia [54]. In our studies, both *SRY* and *SOX9* over-expression were able to induce *NWD1* expression *in vitro*, consistent with the presence of predicted binding sites for these transcription factors in the *NWD1* gene. However, further studies are required to determine whether SRY and SOX9 directly regulate expression of *NWD1* under physiological conditions.

We discovered that both mRNA and protein levels of NWD1 are elevated during prostate cancer pathogenesis or progression. For example, mRNA analysis showed an increase of over 5-fold in *NWD1* expression in stage II prostate cancers compared to normal prostatic glandular epithelium. Upregulation of NWD1 was also observed in some cases of BPH and in PIN lesions, suggesting that altered expression can occur in non-malignant hyperplastic and pre-malignant conditions affecting the prostate glands. Interestingly, NWD1 protein as assessed by immunostaining was strikingly higher in histologically more advanced primary PCa tumors (Gleason grade 3-5 versus Gleason 2). Also, in locally advanced PCa tumors, NWD1 immunostaining was higher in primary tumors that failed to achieve pathological complete response (pCR) to anti-androgen treatment in the neoadjuvant setting [55, 56]. If confirmed in independent patient cohorts, assessment of NWD1 expression levels may provide a biomarker for predicting patient prognosis in some clinical contexts.

While experimental manipulation of *NWD1* expression in PCa and other types of tumor cell lines had some modest effects on NF-κB activity and cellular sensitivity to cytotoxic cytokines and DNA-damaging anticancer drugs, the most striking cellular phenotype that we uncovered for NWD1 was the profound dependence of LNCaP cells on NWD1 for their growth and survival in culture. LNCaP are the most androgen-dependent of the available PCa cell lines, which may explain why they were particularly sensitive to loss of NWD1. Consistent with this hypothesis, we determined that NWD1 modulates steady-state levels of AR in PCa cells, possibly by affecting AR protein stability. A positive correlation between NWD1 and AR protein levels was confirmed both *in vivo* and *in vitro*. Consistent with the requirement for NWD1 for maintaining AR protein levels, we also determined that NWD1 depletion negatively modulates *PSA* expression (an endogenous AR-responsive gene) and significantly decreases androgen-dependent induction of AR-responsive reporter genes (driven by *PSA* and *MMTV* promoters).

An additional cellular function for NWD1 was uncovered by transcriptome profiling, using PPC-1 cells in which *NWD1* expression was silenced. In this regard, we determined that expression of Prostate-derived Ets factor (PDEF) is NWD1-dependent in all human cell lines here tested. Ets transcription factors play an important role in a number of cellular processes including regulation of cell differentiation, proliferation, angiogenesis, and apoptosis. PDEF was originally described as an mRNA transcript highly expressed in prostate tumor cells where it regulates *PSA* expression, acting as an AR co-regulator [30]. In fact, the first PDEF binding partner reported was AR. However, some contradiction is found in the literature regarding the role of PDEF in PCa, where some groups have reported loss of PDEF during tumor progression [57, 58], while others showed increased expression of PDEF in PCa in comparison to BPH and PIN [31]. Still, *PDEF* expression is reportedly induced via the AR pathway [30]. Moreover, Nkx3.1, a prostate tumor suppressor that is frequently lost in PCa, is also a PDEF inhibitor [59], which would be more consistent with an oncogenic role for PDEF in PCa.

The dependence of *PDEF* expression on NWD1 in a variety of tumor cell lines therefore suggests another connection of NWD1 to AR signaling pathways. How precisely NWD1 exerts its effects on AR and PDEF remains to be determined, but our proteomic analysis of NWD1-interacting proteins suggests an association with molecular chaperone-containing protein complexes. In this regard, molecular chaperones are seminal players throughout the life cycle of AR, since they are known to bind AR and to modulate its folding, activation, trafficking, and transcriptional activity [60]. Also, the domain structure of NWD1 suggests that this protein could act as a scaffold and/or co-chaperone molecule, in as much as WD40 repeats facilitate protein–protein interactions and thereby coordinate multi-protein complex assemblies [61]. Therefore, NWD1 presumably exerts its effect in the cytoplasm where we found this protein to be predominantly localized. Thus, the site of action of NWD1 may be manifested at times when AR and other unknown NWD1 target proteins are in the cytosol.

In conclusion, based on the data available thus far, we hypothesize a signaling mechanism (Fig. 6) whereby NWD1 acts downstream of SRY family transcription factors to modulate AR activity by stabilizing AR protein levels and by promoting expression of the AR co-activator PDEF in prostate cells. These events would be anticipated to culminate in higher levels of AR activity and thereby aid prostate cell proliferation, growth, and survival. A better understanding of the molecular and cellular properties of NWD1 and the mechanisms by which it modulates AR signaling, as well as the complete framework of NWD1-interacting proteins, merit further investigation to strengthen potential applications of NWD1 as a possible biomarker or therapeutic target for PCa. Indeed, with regards to therapeutic opportunities, the nucleotide-binding NACHT domain of NWD1 theoretically may provide opportunities for small molecule drug discovery based on searches for compounds that displace directly or allosterically nucleotides, which are cofactors required for oligomerization and activation of other NLR family proteins [62, 63].

## MATERIALS AND METHODS

### Cell lines, reagents and antibodies

Human embryonic kidney (HEK) 293T and cervical carcinoma HeLa-S cell lines were maintained in Dulbecco modified Eagle medium (DMEM) (CellGro, Mediatech) supplemented with 10% heat-inactivated fetal bovine serum (Hyclone, Thermo Scientific) and 1% antibiotic/antimycotic solution (Omega Scientific) at 37°C in an atmosphere of 10% and 5% CO_2_, respectively. All prostate cell lines and THP.1 monocytic cells were cultured in RPMI 1640 medium (CellGro, Mediatech) with the same supplements, at 37°C in an atmosphere of 5% CO_2_.

The transfection reagents Fugene HD™ (Roche Applied Science), GenJet™ (SignaGen Laboratories), JetPrime™ (Polyplus Transfection) and TransIT-2020 (Mirus Bio) were acquired from the manufacturers. The synthetic androgen R1881 (methyltrienolone), doxorubicin, taxol (paclitaxel) and TNF-α were obtained from Sigma-Aldrich, and TRAIL was from Calbiochem (EMD Chemicals).

The rabbit anti-NWD1 (585) antibody was raised by immunization with specific antigenic peptide (N-LITLPLVGKPLNLDRKVAPQ-C) (Lampire Biological Laboratories). The rabbit anti-AR (N-20) and mouse anti-ubiquitin (P4D1) antibodies were purchased from Santa Cruz Biotechnology. Mouse anti-FLAG affinity gel (catalog no. A2220-5ML), anti-FLAG (catalog no. F3165), anti-β-actin (catalog no. A5441) and anti-α-tubulin (catalog no. T9026) antibodies were purchased from Sigma-Aldrich. Rabbit anti-PARP (catalog no. 9542) antibody was from Cell Signaling Technology. The IRDye™ secondary antibodies were purchased from LI-COR Biosciences.

### Cloning and lentivirus production

The human *NWD1* open reading frame (ORF) was isolated by RT-PCR (Expand High Fidelity PCR System, Roche) from a pooled cDNA library derived from several organs (fetal brain and kidney, testis, placenta, prostate and breast). Gene-specific primers were designed based on its predicted cDNA sequence (GenBank BC117698.1). The FLAG-tagged *NWD1* cDNA was generated after cloning into pcDNA3-Flag plasmid (provided by Shu-ichi Matsuzawa, SBMRI, CA, USA).

For the generation of shRNA-expressing constructs and lentiviral particles, single 83-mer oligonucleotides were designed, containing an XbaI site at the 5’ end and sense and antisense shRNA strands intermediated by a short spacer, plus a partial sequence of the H1-RNA promoter at the 3’ end. Standard PCR procedures (Advantage 2 PCR kit, Clontech) were performed using specific shRNA oligonucleotides and T3 primer plus pSuper-like plasmid [64] as a template to provide H1 promoter driven shRNA cassettes with an additional XbaI site at the 3’ end. PCR products were purified (Qiagen), digested with XbaI, and cloned into the 3’ LTR NheI site of a CMV-GFP lentiviral vector as described (1). The LV-shGFP construct (control) was kindly donated by Oded Singer (Salk Institute, CA, USA). The following shRNA oligonucleotides were used: 5’- CTGTCTAGACAAAAAACACATCCTTGAAGACTGCTCTCTTGAAGCAGTCTTCAAGGATGTG TGGGGATCTGTGGTCTCATACA-3’ for shNWD1_1; and 5’- CTGTCTAGACAAAAAGGATGACAAGTATGTGTACTCTCTTGAAGTACACATACTTGTCATCCGGGGATCTGT GGTCTCATACA -3’ for shNWD1_2. Vesicular stomatitis virus G envelope protein-pseudotyped lentiviruses were prepared and purified as described [65].

### RNA analysis

Total RNA was extracted using RNeasy™ Plus Mini kit (Qiagen). After isolation, 1–5 μg total RNA was reverse-transcribed, in the presence of oligo(dT) primer, according to the manufacturer (SuperScript First-Strand Synthesis, Invitrogen). First-strand cDNA was diluted and analyzed in triplicate with gene-specific primers by real-time PCR, using a Stratagene Mx3000p sequence detection system with SYBR Green PCR master mix (Applied Biosystems). Gene expression data (fold induction) were normalized with the respective levels of 18S ribosomal RNA or cyclophilin (CPH) mRNA. The Human Major Tissue qPCR Array (HMRT102) and the TissueScan Prostate Cancer Tissue qPCR Panel I (HPRT101) were normalized by the manufacturer (Origene Technologies, Inc.) and quantitatively screened using specific primers for human NWD1 (forward: 5’-CAATGTGCTTGCTTCTCCAA-3’; reverse: 5’-CTGGTGGTTGGGATGATTCT-3’). Additional primer sequences are available upon request.

For gene expression microarray analysis, labeled cRNA was prepared from 500 ng RNA using the Illumina™ RNA Amplification Kit (Ambion). The labeled cRNA (1500 ng) was hybridized overnight at 58°C to the Sentrix™ HumanWG-6 Expression BeadChip (>46,000 gene transcripts; Illumina) according to the manufacturer’s instructions. BeadChips were subsequently washed and developed with fluorolink streptavidin- Cy3 (GE Healthcare). BeadChips were scanned with an Illumina™ BeadArray Reader. The generated data was acquired and analyzed using the BeadStudio (Illumina) and Ingenuity IPA™ software (Ingenuity Systems). All microarray data has been deposited in GEO (accession number GSE53115).

### Immunohistochemistry

Prostate cancer tissue microarrays (TMAs) were constructed as described [66]. An IRB-approved protocol was used to retrieve archival paraffin blocks from the Department of Urology, University of California, Irvine, USA for the UCI NCI SPECS consortium [67]. The TMAs included transurethral resection of the prostate (TURP) and radical prostatectomy specimens from 109 patients with locally confined (stage T2) and locally advanced disease (stage T3). In addition, 34 selected blocks were derived under IRB approval from Department of Pathology, University of California, San Diego, representing tumors from 18 prostatectomy specimens from patients who received preoperative hormone treatment and whose specimens showed histologic evidence of either no or partial response, suggesting hormone refractoriness. The tumor specimens were organized in the TMA format.

Dewaxed tissue sections were immunostained as reported previously [68] using rabbit polyclonal antibodies to AR and NWD1. Application of the primary antibody was followed by incubation with goat anti-rabbit polymer-based EnVision-HRP-enzyme conjugate (Dako Cytomation). DAB chromogen (Dako Cytomation) was applied, yielding brown color. The TMA slides were scanned at an absolute magnification of 400x [resolution of 0.^25^ mm/pixel (100,000 pix/in.)] using the Aperio ScanScope CS1 (Aperio Technologies). The acquired digital images were evaluated for NWD1 and AR immunopositivity. The NWD1 immunostaining results were arbitrarily scored according to intensity as 0, negative; 1+, weak; 2+, moderate; or 3+, strong. The scoring was based on the percentage of immunopositive cells (0 to 100) multiplied by staining intensity score (0/1/2/3), yielding scores of 0 to 300. Data were analyzed using the STATISTICA software package (StatSoft). A one-way analysis of variance (ANOVA) method was used to determine the significance of differences in the NWD1 protein distribution among the different histological categories, and among the cancers with different Gleason grades, or those partially responding or not responding to anti-androgen therapy.

### Cell viability assays

Cells in 96-well plates were analyzed for mitochondrial dehydrogenase activity (as a means of cell proliferation/viability status) using the WST-1 assay according to the manufacturer’s protocol (Roche). In brief, 10 μL of WST-1 reagent was added to 100 μL of cell medium and incubated for 1–2 h at room temperature. The formazan dye formed was measured at 450 nm and 690 nm (background) using a FlexStation 3 Microplate Reader (Molecular Devices).

Cell viability was microscopically assessed after crystal violet staining. Briefly, culture dishes were washed three times with phosphate-buffered saline (PBS), and remaining adherent cells were fixed and stained with 0.1% crystal violet solution in 20% methanol.

### Protein analysis

For immunoprecipitation (IP) applications, cells were lysed with IP buffer (20 mM HEPES [pH 7.^5^], 150 mM NaCl, 5mM MgCl_2_, 2 mM EDTA 1% NP-40, 10% glycerol) supplemented with 1 mM dithiothreitol (DTT) and protease inhibitor cocktail (Roche). Equal amounts of clarified protein lysates (2-4 mg) were incubated with 20-30 μl of anti-FLAG™ M2 affinity gel (Sigma), in 1 ml of IP buffer for 12 to 16 hours at 4°C. Immunoprecipitates were washed three times with same buffer, one last wash with TBS buffer (20 mM Tris-HCl pH 7.5, 150 mM NaCl) and eluted with 0.5 mg/ml FLAG-tag peptide (Sigma) in TBS buffer.

For sub-cellular fractionation, cytosolic fractions were obtained by extensive resuspension of cell pellets into buffer A (10 mM Hepes 7.5, 1.5 mM MgCl_2_, 10 mM KCl, 0.5 mM DTT and protease inhibitors). The remaining pellet was extensively resuspended into buffer C (20 mM Hepes 7.5, 25% glycerol, 450 mM NaCl, 1.5 mM MgCl_2_, 0.2 mM EDTA and protease inhibitors) to isolate the nuclear sub-fraction. Total lysates and subcellular fractions were quantified before SDS-PAGE procedures (Bio-Rad Protein Assay). Immunoblotting was performed using various antibodies.

### Reporter gene assays

For reporter gene assays, cells were transduced with a lentiviral vector to introduce 5×κB-driven luciferase reporter gene or transfected with PSA- or MMTV-mediated luciferase reporter gene, TK-mediated Renilla plasmid and respective amounts of testing vectors. Cells were plated as indicated and treated with respective inducers for defined periods of time. Luciferase activity was measured as suggested by manufacturer’s protocol (Steady-Glo™ or Dual-Luciferase™ Reporter Assay Systems, Promega), using a FlexStation 3 Microplate Reader (Molecular Devices).

### Mass spectrometry (LC-MS/MS)

IP eluates were adjusted to 200 μl with 50 mM ammonium bicarbonate. Then, 4 ul Tris(2-carboxyethyl)phosphine (TCEP) was added to each sample and proteins were reduced at 60°C for 30 min. Iodoacetamide was added (to 20 mM) and proteins were alkylated at 30 min at room temperature in the dark. Mass spectrometry grade trypsin (Promega) was added (1:20 ratio) for overnight digestion at 37°C. After digestion, formic acid was added to the peptide solution (to 2%), followed by desalting with Microtrap (Thermo Scientific) and then online analysis of peptides by high-resolution and high-accuracy LC-MS/MS consisting of a Michrom HPLC, a 15-cm Michrom Magic C18 column, a low-flow ADVANCED Michrom MS source, and a LTQ-Orbitrap XL (Thermo Fisher Scientific). A 120 min gradient of 10–30%B (0.1% formic acid, 100% acetonitrile) was used to separate the peptides. The total LC time was 140 min. The LTQ-Orbitrap XL was set to scan precursors in the Orbitrap followed by data-dependent MS/MS of the top 10 precursors.

The LC-MSMS raw data were submitted to Sorcerer Enterprise v.3.5 release (Sage-N Research Inc.) with SEQUEST algorithm as the search program for peptide/protein identification. The differential spectral count analysis and proteolytic profile mapping was performed by QTools, an open source in-house tool developed for automated differential peptide/protein spectral count analysis and Gene Ontology [69].

## SUPPLEMENTARY FIGURES


