# Supplementary material for: The NLR-related protein NWD1 is associated with prostate cancer and modulates androgen receptor signaling
Source: Oncotarget. 2014 Mar 26;5(6):1666–82. doi: 10.18632/oncotarget.1850 (PMC4039239; doi:10.18632/oncotarget.1850)
Supplement: Supplementary file 1 [file oncotarget-05-1666-s001.pdf]

The NLR-related protein NWD1 is associated with prostate cancer and modulates androgen receptor signaling

SUPPLEMENTARY FIGURE LEGENDS

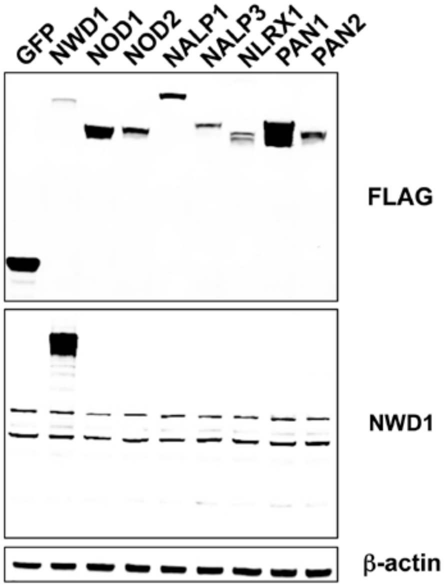

Supplemental Figure 1. Specificity profile of anti-human NWD1 antibody. HEK 293T cells in 10-centimeter plates (90% cell confluency) were transfected by calcium phosphate with 20 μg of a series of FLAG-tagged constructs (as indicated). At 48 hours post-transfection, cells were lysed and 24 μg of protein lysate was resolved by SDS-PAGE. Immunoblotting analysis was performed using antibodies for FLAG, NWD1 (585) and β-actin (loading control).

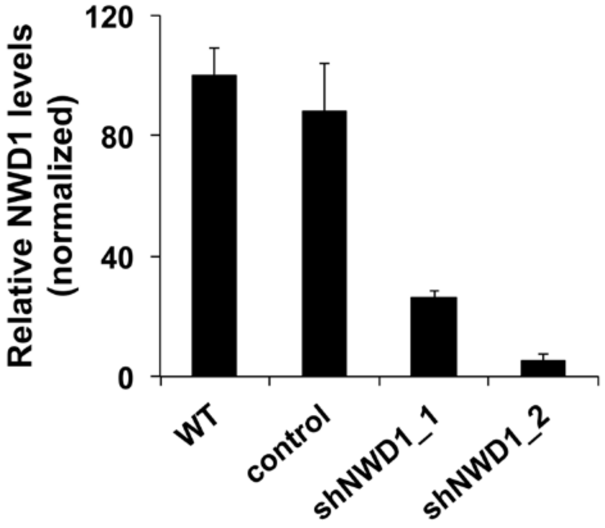

Supplemental Figure 2. Validation of *NWD1* silencing by Real-Time PCR. Transduced PPC-1 cells constitutively expressing shRNAs against GFP (control) or NWD1 (shNWD1_1 and shNWD1_2) were processed for isolation of total RNA and first-strand cDNA synthesis. Non-transduced wild-type (WT) cells were also processed as a control. RNA levels were normalized by cyclophilin (CPH) expression. Relative values (y-axis) were obtained by comparison to the expression in wild-type cells (mean value = 100). The values presented are averages of at least three replicates ($\pm$ standard deviation).

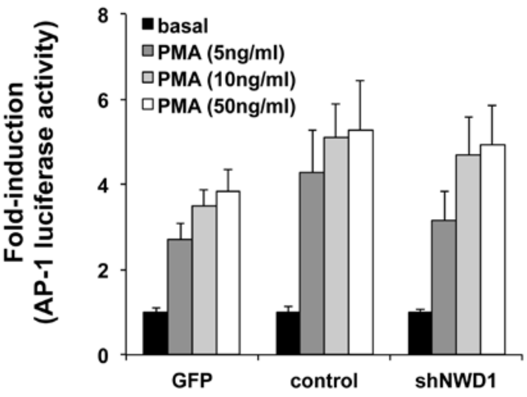

Supplemental Figure 3. Activity towards AP-1 signaling pathway. HEK 293T cells stably constitutively expressing GFP, shRNAs for GFP (control) or NWD1 (shNWD1) were plated in 10-cm dish at same cell density and, at 80% confluency, were transfected by calcium phosphate with 18.5 μg of AP-1 mediated firefly luciferase and 4 μg thymidine kinase (TK)-mediated Renilla reporter plasmids. At 36 hours post-transfection, cells were resuspended and loaded into 96-well plate at 10^5 cells/well. Cells were further treated with different concentrations of Phorbol 12-myristate 13-acetate (PMA) for 20 hours. Luciferase activity was measured using Dual-Luciferase™ Reporter Assay System (Promega). Values were normalized according to Renilla luciferase signals. Values converted to fold-induction in AP-1-mediated activity according to the measurement obtained for respective non-treated (basal) cells (mean value=1). The values presented are averages of three replicates (± standard deviation).

Top Networks

ID	Associated Network Functions	Score
1	Lymphoid Tissue Structure and Development, Tissue Morphology, Cell-To-Cell Signaling and Interaction	44
2	Cancer, Cell Death, Neurological Disease	42
3	Cancer, Cell-To-Cell Signaling and Interaction, Gastrointestinal Disease	35
4	Gene Expression, Cell Morphology, Cellular Assembly and Organization	35
5	Organ Development, Organ Morphology, Visual System Development and Function	34

Top Molecules

Fold Change up-regulated		Fold Change down-regulated	
ITGB1*	↑2.496	RPPH1 (includes EG:85495)	↓-4.005
ACTG1*	↑2.315	MT2A	↓-3.675
PLAU	↑2.142	BCYRN1	↓-3.374
SC4MOL	↑2.135	MT1A	↓-3.207
PSMC4*	↑2.122	CLDN10	↓-2.391
CTNNB1	↑2.092	SLPI	↓-2.390
SLC3A2*	↑2.091	HIST2H2AA3*	↓-2.306
XPOT	↑2.061	SEPW1	↓-2.234
TCEA1	↑2.059	CATSPER2	↓-2.211
GNG12	↑1.954	C4BPB	↓-2.184

Supplemental Figure 4. Networks and molecules involved in NWD1 signaling. Comparative gene expression microarray data was analyzed systematically by Ingenuity Pathway Analysis (IPA) to identify potential interaction networks based on NWD1 regulated genes. The biological functions of the highest scoring networks correlated with *NWD1* expression are shown on the top panel. Genes whose expression was altered after *NWD1* knockdown in PPC-1 cells ($\geq$ 2-fold change) are shown on the bottom panel.

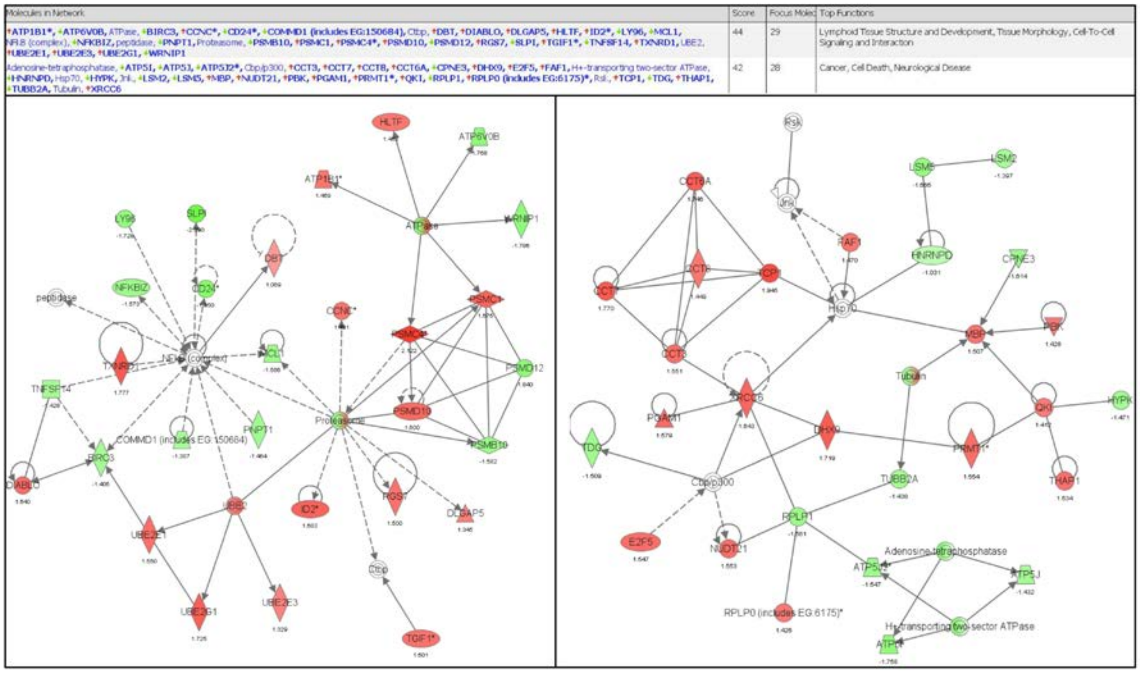

Supplemental Figure 5. Construction of functional interaction network using potential NWD1 modulated genes. Snapshot of top networks with most abundant number of differentially expressed genes (≥ 1.3-fold change) after *NWD1* knockdown. The respective molecules involved on each network as well as the function of these networks are listed on the top panel. The connection among these target molecules to further integrate each signal transduction network is shown on the bottom panels. Up- and down-regulated genes are shown in red and green shapes, respectively. The fold-change on gene expression for each target molecule (after NWD1 depletion) is also indicated.

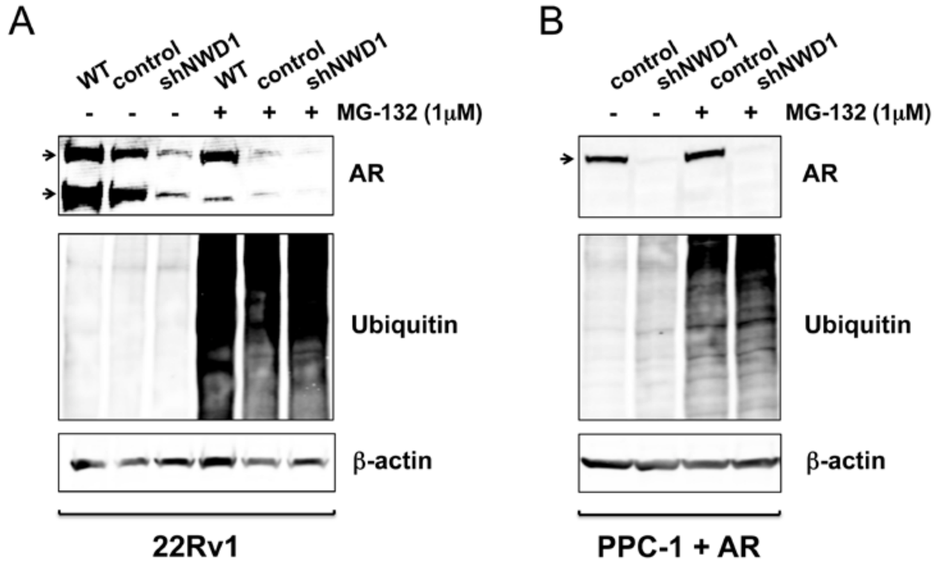

Supplemental Figure 6. MG132 does not restore AR levels in NWD1-depleted PCa cells. (A) Wild-type (WT) or transduced 22Rv1 cells, stably expressing shGFP (control) or shNWD1, were cultured in charcoal-stripped serum containing medium (6-well dish, 90% confluency) for 48 hours and further treated with (+) or without (-) 1 μM MG-132 for 26 hours. (B) PPC-1 cells in 6-well dish (80% confluency) were transfected by Fugene HD™ (Roche) with 2.2 μg of shGFP (control) or shNWD1 constructs plus 1.1 μg of CMV-mediated AR expression vector (3.3 μg DNA per well). At 12 hours post-transfection, cells were cultured in charcoal-stripped serum containing medium for 48 hours and further treated with (+) or without (-) 1 μM MG-132 for 26 hours. Total cell lysates were isolated and analyzed (50 μg protein) by immunoblotting using antibodies specific for AR, ubiquitin and β-actin (loading control). AR protein bands are indicated by arrows.

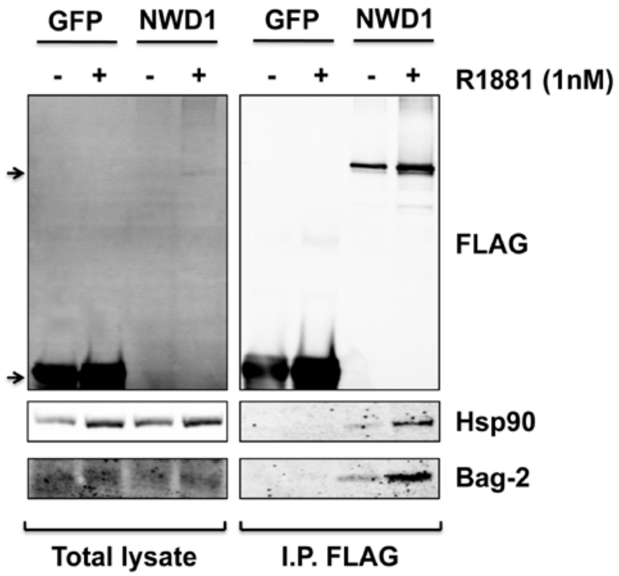

Supplemental Figure 7. Validation of Hsp90 and Bag-2 as NWD1 interacting proteins. LNCaP cells in T-75 flasks (70% confluency) were transfected by Fugene HD^{TM} (Roche) with 11 μg of FLAG-GFP (control) or FLAG-NWD1 expression constructs. At 48 hours post-transfection, cells were cultured in charcoal-stripped serum containing medium for further 48 hours and then treated with (+) or without (-) 1 nM R1881 for 30 hours. Total cell lysates (50 μg protein, left panel) and FLAG immunoprecipitates (right panel) were analyzed by immunoblotting using antibodies specific for FLAG, Hsp90 and Bag-2. FLAG-tagged proteins, detected in the total lysates, are indicated (arrows).
